# The Emerging Role of Neutrophil Extracellular Traps in Arterial, Venous and Cancer-Associated Thrombosis

**DOI:** 10.3389/fcvm.2021.786387

**Published:** 2021-12-02

**Authors:** Yilu Zhou, Weimin Tao, Fuyi Shen, Weijia Du, Zhendong Xu, Zhiqiang Liu

**Affiliations:** Shanghai First Maternity and Infant Hospital, School of Medicine, Tongji University, Shanghai, China

**Keywords:** neutrophil, neutrophil extracellular traps, thrombosis, deep vein thrombosis (DVT), cancer thrombosis

## Abstract

Neutrophils play a vital role in the formation of arterial, venous and cancer-related thrombosis. Recent studies have shown that in a process known as NETosis, neutrophils release proteins and enzymes complexed to DNA fibers, collectively called neutrophil extracellular traps (NETs). Although NETs were originally described as a way for the host to capture and kill bacteria, current knowledge indicates that NETs also play an important role in thrombosis. According to recent studies, the destruction of vascular microenvironmental homeostasis and excessive NET formation lead to pathological thrombosis. *In vitro* experiments have found that NETs provide skeletal support for platelets, red blood cells and procoagulant molecules to promote thrombosis. The protein components contained in NETs activate the endogenous coagulation pathway to promote thrombosis. Therefore, NETs play an important role in the formation of arterial thrombosis, venous thrombosis and cancer-related thrombosis. This review will systematically summarize and explain the study of NETs in thrombosis in animal models and *in vivo* experiments to provide new targets for thrombosis prevention and treatment.

## Introduction

The role of neutrophil extracellular traps (NETs) in inflammation and thrombosis has been controversial for decades (1, 2). Traditionally, thrombosis is considered only a blood vessel or blood disease (2). Inflammation and thrombosis are two independent pathological processes (3). However, with the progress of immunology research, researchers have discovered that thrombosis is an inflammatory process (4). A previous study observed neutrophil exudation in the blood vessel wall at the early stage of thrombosis induction, followed by monocytes and lymphocytes (5). Based on accumulating evidence, neutrophils play a key role in the process of thrombosis (5–8). The depletion of neutrophils has been shown to reverse experimental thrombosis (9, 10). Another study reported that neutrophils produce tissue factor (TF) and contribute to the formation of thrombosis *in vivo* and *in vitro* (11).

In 2004, Brinkmann et al. found that neutrophils form a structure that separates from the cell itself after stimulation called neutrophil extracellular traps (NETs), which provided new ideas for studies of the interaction between neutrophils in the pathway of inflammation and thrombosis (12). NETs were discovered as extracellular strands of decondensed DNA in complex with histones and granule proteins, which were expelled from dying neutrophils. NETs are composed of circulating markers (myeloperoxidase, neutrophil elastase, etc.) and kill bacteria during general inflammation.

**Figure 1:**
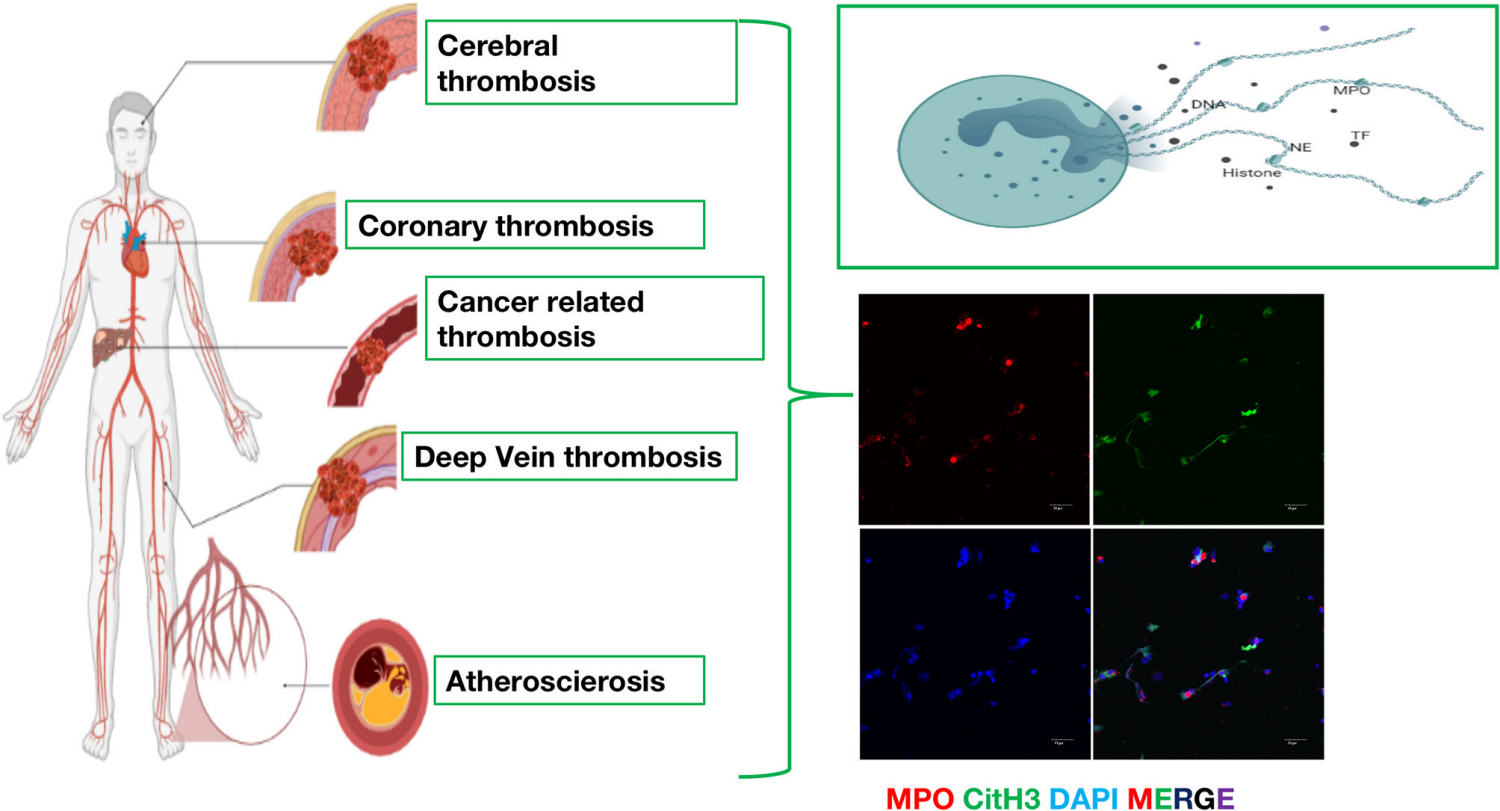
The emerging role of neutrophil extracellular traps (NETs) in arterial thrombosis, venous thrombosis and cancer-related thrombosis. Immunofluorescence of NETs. Red: MPO, Green: CitH3, Blue: DAPI.

Additionally, NETs play vital roles in arterial, venous and cancer-related thrombosis formation. For arterial and venous thrombosis, activated components are composed of DNA and histones, which provide a structure for red cells and platelets and contribute to the formation of thrombosis. For cancer-related thrombosis, tumor cells can stimulate the formation of NETs and then promote the metastasis of cancer. Therefore, understanding the different mechanisms of thrombosis has become an important direction for the study of thrombotic diseases (Figure 1). The relevant research progress in recent years is summarized below.

## The Role of NETs In Atherosclerosis and Arterial Thrombosis

Chronic inflammation plays an important role in the occurrence and development of atherosclerosis and thrombosis, during which the status of neutrophils has received increasing attention (13). Many studies have confirmed an important role for NETs in the processes of atherosclerosis, coronary artery disease (CAD) and ischemic stroke (14–16). However, most of these studies are based on *in vitro* and animal experiments (Table 1). Few studies have assessed the clinical value of NETs in arterial thrombosis.

**Table 1:** NETs in atherosclerosis and arterial thrombosis.

**Objects**	**Main findings**	**References**
Patients with STEMI	NETs were dominated in early thrombosis	(17)
Patients with AMI or AIS	The formation and abundance of NETs were associated with prognosis	(18)
Patients with STEMI	Plasma levels of NETs markers increased and were positively correlated with infarct size and left ventricular dysfunction	(19)
Patients with COVID-19	NETs were present in coronary thrombus of patients with COVID-19 and myocardial infarction	(20)
Patients with diabetes	Elevated levels of NETs markers (e.g., citH3) were associated with severe coronary atherosclerosis in patients with diabetes	(21)
Patients with CAD or AIS	citH3 was observed in almost all thrombi	(2)
Mouse/atherosclerosis	NETs lysed smooth muscle cells, leading to the destabilization of plaques.	(22)
Mouse/diabetes	NETs promoted macrophage inflammation and inhibited atherosclerosis resolution	(23)
Integrin 1α^−/−^ mouse	Decreased levels of NETs resulted in decreased platelet aggregation, cathepsin-G secretion, and arterial thrombosis	(24)

*NETs, neutrophil extracellular traps; STEMI, ST-elevation myocardial infarction; AMI, acute myocardial infarction; AIS, acute ischemic stroke; CAD, coronary artery disease*.

### NETs in Atherosclerosis

**Figure 2:**
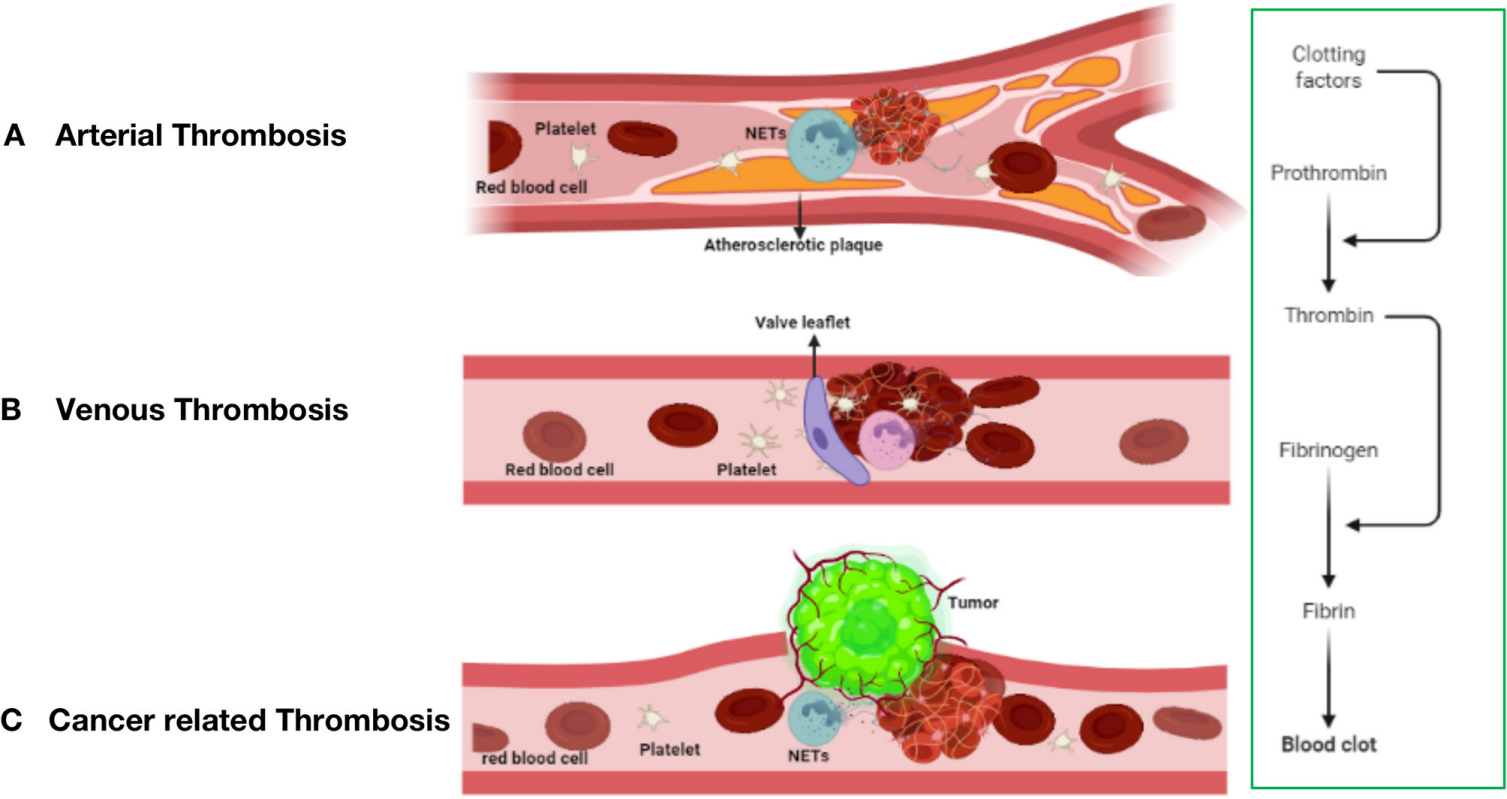
The mechanism of thrombosis formation in arterial thrombosis **(A)**, venous thrombosis **(B)**, and cancer-related thrombosis **(C)**.

NETs have been detected in patients with atherosclerosis and in animal models, and NETs are related to various pathogeneses of atherosclerosis. NETs play a role in all stages of atherosclerosis, from early endothelial dysfunction to atherosclerotic plaque rupture and atherosclerotic thrombosis (25, 26). As a scaffold for cells and various coagulation factors, NETs not only exist in plaques and thrombi but also induce oxidative stress, induce the activation of endothelial cells, antigen-presenting cells and platelets, increase the expression of coagulation factors, and lead to proinflammatory reactions (Figure 2A). These structures play a role in the pathogenesis of atherosclerotic plaque formation and thrombosis (3, 4, 27). Compared to the network structures produced by other cells, NETs are mainly produced in the early stage of thrombosis, and most of them are formed in the acute stage of the disease (17, 28). NETs also induce the death of smooth muscle cells, leading to reduced plaque stability (22). At the same time, NETs also promote the abnormal activation of macrophages and upregulate the levels of IL-8 and inflammasomes, thereby further amplifying the role of NETs and accelerating the progression of atherosclerosis (23, 29–31). Macrophages are more common in the types of lesions that are prone to rupture, but studies have shown that the infiltration of neutrophils is more important for these erosion-prone lesions (32). Oxidized low-density lipoprotein (oxLDL), which easily accumulates in macrophages, is used as an atherosclerosis-inducing molecule. Many studies have shown that it stimulates neutrophils to form NETs (33). The specific deletion of peptidyl arginine deiminase-4 (PAD4) reduces the formation of NETs and significantly alleviates atherosclerosis, complications, and inflammation caused by macrophages (34, 35).

### NETs in Coronary Thrombosis

NETs promote coronary microvascular thrombosis and affect heart function (36). The content of NETs in cardiogenic thrombosis is higher than that in thrombosis with other causes (37). NETs carrying tissue factor are often observed at the site of coronary thrombosis, and studies have shown that NETs are a potential marker of arterial thrombosis in clinical specimens and animal models (38). The structure of NETs has been detected in thrombi of ST-segment elevation myocardial infarction (STEMI) and non-ST-segment elevation myocardial infarction (NSTEMI) (39), and the myocardial infarction score (18) and circulating markers (citrullinated histone 3; myeloperoxidase, neutrophil elastase, etc.) of NETs are significantly reduced after treatment (40). The activation of platelets and neutrophils increases the risk of major adverse cardiovascular events (MACEs) after acute myocardial infarction (41). Moreover, NETs stimulate fibroblasts, affect heart remodeling after STEMI and are important mediators of fiber remodeling (19). Recent studies have also shown a major role for NETs in the onset of STEMI in patients with COVID-19, and NETs have been detected in thrombus samples from all patients with COVID-19 (20).

### NETs in Cerebral Thrombosis

NETs are also an important component of cerebral thrombosis, are cytotoxic to endothelial cells, and together with von Willebrand factor (VWF), promote the hypercoagulable state (42, 43). Abundant NET structures have been observed in almost all thrombi removed from patients with ischemic stroke (37, 44), and patients with cardiogenic embolism have higher levels of NETs (45). In cerebral ischemia, neutrophils are the first type of cells that migrate to damaged brain tissue. They produce NETs in the brain parenchyma and cerebral blood vessels, thereby aggravating inflammation and brain tissue damage (46). Accumulating evidence indicates that NETs may fight against tissue-type plasminogen activator (t-PA) by promoting coagulation and stabilizing clot-induced thrombosis, which is a problem often encountered in the treatment of patients with stroke (47–49). NETs may be potential biomarkers and therapeutic targets for recurrent stroke in patients with severe carotid artery stenosis (50). Deoxyribonuclease (DNase) degradation of NETs or treatment with PAD4 inhibitors to prevent NET formation significantly inhibits arterial thrombosis in the brains of ischemic mice and improves stroke prognosis, such as by reducing the site of infarction involvement and maintaining basic blood flow levels (51).

### NETs as Circulating Markers of Arterial Thrombotic Diseases

The components of various NETs in plasma have been suggested to be used to predict the severity of diseases such as CAD and ischemic stroke. For example, PAD4 levels are very high in carotid plaques (52), and citrullinated histone H3 (CitH3), double-stranded DNA (dsDNA), neutrophil elastase (NE) levels, the myocardial infarction area, and left ventricular dysfunction are related to the poor prognosis of patients with coronary atherosclerosis and myocardial infarction (19, 21, 41). The plasma levels of the MPO-DNA complex and cell-free DNA (cfDNA) are directly proportional to immune thrombosis in patients with COVID-19 complicated with acute respiratory distress syndrome (6, 53). NET-related tissue factors may also be used as markers (54). In patients with type 2 diabetes, circulating markers of NETs are related to thrombosis and a low fibrinolytic status and can be used as biomarkers for the stratification of patients with diabetes who present a higher risk of vascular complications (55, 56). Immunofluorescence staining showed the presence of NETs in thrombus samples from animal models. In integrin1α^−/−^ mice, the proportion of neutrophils releasing NETs is reduced, and arterial thrombosis is significantly reduced (24). In the mouse myocardial infarction model, the inhibition of PAD4 activity by an intraperitoneal injection of a specific drug reduces the infarct size and improves the prognosis of cardiac ischemia (57).

### The Therapeutic Potential of DNase in Patients With Arterial Thrombosis

Lower DNase activity is related to infarct size (58), and increased DNase activity reduces the risk of host tissue damage and thrombosis induced by NETs (59). DNase treatment reduces the content of NETs in plaques and the level of macrophage inflammation, promotes disease remission and improves prognosis (23). The use of t-PA for thrombolytic therapy is the basis for the treatment of thrombotic diseases. The addition of DNase targeting NETs to the standard t-PA treatment regimen increases the therapeutic effect of thrombolytic therapy and improves the prognosis of the disease (37, 51). Taken together, these data indicate that DNase improves the prognosis of subjects with cardiac and cerebral ischemia (37, 51). The role of NETs in arterial thrombosis indicates that DNase degradation of NETs may become a new treatment direction and improve the effectiveness of thrombolytic therapy. In the future, large-scale research should use NETs and their components as disease markers and potential therapeutic targets to reduce atherosclerosis and prevent thrombosis. At the same time, further research is needed to explore the effectiveness and safety of DNase in the treatment of arterial thrombotic diseases.

## The Role of NETs in Venous Thrombosis

Unlike arterial thrombosis, venous thrombosis is not caused by endothelial rupture and is mostly due to slow venous blood flow (Figure 2B), a hypercoagulable blood state and venous intima injury (60, 61). Prolonged lack of exercise, pregnancy, and chronic venous blood supply are the main causes of impaired venous blood flow and are associated with an increased risk of DVT development (62). Venous thrombosis is rich in fibrin and red blood cells, and a large amount of white blood cell infiltration is observed (63).

### NETs in Deep Vein Thrombosis

The annual incidence of venous thromboembolism (VTE) in the United States is ~1/1000 (64). Stasis of blood flow in the veins is one of the main causes of DVT, and this process often leads to immune thrombosis. A study compared 150 symptomatic patients with DVT with a control group that was clinically suspected of having DVT but had a negative objective test and found that compared with the control group, patients with DVT had higher levels of circulating nucleosomes and activated neutrophils. The increase in the levels of the two parameters indicates that the risk of DVT is increased approximately 3 times (65). Deep vein thrombosis (DVT) is the most common complication in patients with traumatic fractures. In patients with traumatic fractures, the levels of citrullinated histone H3 (H3Cit), cfDNA and nucleosome NET biomarkers in plasma were detected, and H3Cit and cfDNA assisted in the diagnosis of DVT in patients with traumatic fractures (66). The thrombus in a patient with microscopic polyangiitis (MPA) complicated with deep vein thrombosis (DVT) was confirmed to be rich in neutrophils (67). Furthermore, NETs exist in the thrombus tissue of patients with venous thromboembolism, which is related to the maturation of human thrombi (61). The formation of NETs may be short-lived, occurring when neutrophils are recruited into the thrombus, and as the thrombus matures, extracellular NETs are degraded. Therefore, NETs are rarely found in a mature thrombus. NETs have been used as human DVT biomarkers (68).

Both NETs and inflammasome activation play a role in the development of DVT. The stimulation of neutrophils induces the formation of NETs and activates Caspase-1. Active Caspase-1 requires NETs as an adhesion surface. NETs and their component histones promote the activation of Caspase-1 in platelets. Colocalized NETs and Caspase-1 and platelet recruitment were observed at the site of thrombosis. Pharmacological inhibition of Caspase-1 substantially reduces DVT in mice, and thrombi are still formed without citrulline histone 3. These data indicate an interaction between NETs and inflammasomes *in vitro* and in the environment of deep vein thrombosis. This interaction may be an important mechanism supporting venous thrombosis (69). IL-1β regulates the recruitment of neutrophils, and the inflammasome mediates the activation and secretion of IL-1β. The NLRP-3 inflammasome mainly acts on Caspase-1 and Caspase-11, leading to the cleavage and activation of IL-1β and IL-18.

The venous endothelium plays an important role in the formation of DVT. Marie-Luise von Brühl et al. confirmed that blood monocytes and neutrophils adhere to the venous endothelium, providing an initial stimulus for the development of DVT (62). This study confirmed that neutrophils are a key trigger for the formation of DVT. Neutrophils form NETs, which induce FXII-dependent coagulation. In addition, platelets can also promote leukocyte accumulation and fibrin formation by enhancing neutrophil-dependent coagulation. Vascular endothelial activation or damage locally activates complement to release allergens and chemokines C3a and C5a. These pathways synergistically trigger the recruitment and activation of platelets, neutrophils, and monocytes.

The role of platelets in venous thrombosis is not as obvious as that in arterial thrombosis. However, thrombocytosis is considered a risk factor for VTE (70). Pathogens and DAMPs stimulate neutrophils to activate the coagulation system, and this interaction mediates immune thrombosis. Platelets release high mobility histone B1 (HMGB1), which triggers the formation of NETs (71, 72).

Mixed lineage kinase-like (MLKL)-driven neutrophil necrosis is related to venous thromboembolism (73). Human inferior vena cava thrombosis is positive for phosphorylated MLKL, and phosphorylated MLKL induces cell necrosis. In mice, MLKL colocalizes with citrulline histone H3, and a genetic defect in MLKL partially prevents clot formation during inferior vena cava ligation in mice. Platelets activated by VTE induce NETosis, resulting in the release of chromatin and DAMPs, which contribute to the formation of clots (74).

Heparin-induced thrombocytopenia (HIT) is an immune-mediated thrombocytopenia associated with a severe prethrombotic state. HIT induction leads to increased neutrophil adhesion to the venous endothelium. In HIT mice, neutrophils migrate in a retrograde manner through a CXCR2-dependent mechanism and accumulate in the thrombus. After PF4 binds to NETs, it compresses itself and resists degradation by DNase. The PF4-NET complex selectively binds to HIT antibodies, further protecting them from nuclease digestion. In HIT mice, inhibition of NET formation by Padi4 gene disruption or DNase treatment limits the size of venous thrombosis. Neutrophil activation promotes HIT venous thrombosis by enhancing neutrophil-endothelial cell adhesion and neutrophil clot infiltration, in which the PF4-NET-HIT antibody complex causes thrombosis to spread (75). Therefore, strategies to prevent venous thrombosis may be to inhibit the adhesion of neutrophil endothelial cells, prevent the recruitment of neutrophil chemokine-dependent neutrophils to thrombi, or inhibit the release of NETs.

Many substances in plasma induce DVT. IFNγ promotes venous thrombosis through the formation of NETs, and NK cells play an important role in this process. The specific consumption of natural killer cells (NK) leads to reduced NET formation and reduced thrombus formation (76). C5a, the most effective chemotactic complement activating fragment, is released after C5 protein lysis and is considered a key determinant of neutrophil recruitment and the activation of thrombosis. In the mouse venous thrombosis model, the weight of the thrombin-antithrombin complex is closely related to C5a, which indicates that the process triggered during thrombosis promotes C5a production. *In vitro*, the catalytic efficiency of plasmin-mediated C5a production far exceeds that of thrombin or factor Xa and is similar to the recognized complement C5 convertase. C5 activated by plasmin mediates the production of the membrane attack complex (MAC) (77). Antiphospholipid antibodies (aPLs) activate neutrophils to release NETs, thereby inducing arterial and venous thrombosis inherent in antiphospholipid syndrome (APS). Compared with healthy volunteers, patients with primary antiphospholipid syndrome (APS) have higher levels of cell-free DNA and NETs in serum and plasma. Freshly isolated neutrophils from patients with APS tend to release high levels of spontaneous NETs. In addition, the serum of patients with APS, as well as IgG purified from patients with APS, stimulates the release of NETs from controlled neutrophils. Human monoclonal aPLs, particularly those targeting β2GPI, also enhance the release of NETs. The APS induction of NET production can be eliminated by the formation of reactive oxygen species and TLR4 inhibitors (78).

In addition to classic thrombosis-related substances, other molecules are also involved in the formation of DVT. Resolvin D4 (RvD4) is an SPM (specialized proresolving mediator) that is enriched at the natural beginning of thrombolysis. After administration, the burden of thrombi is significantly reduced, the infiltration of neutrophils in the thrombus is reduced, the number of monocytes increases, and these cells are in the early stage of apoptosis. The number of cells in the apoptotic state increases. Neutrophils of mice treated with RvD4 are less sensitive to the release of NETs (79). Slc44a2 is a ubiquitous transmembrane protein that has been identified as a receptor for a vascular hematoma factor (VWF). The expression of the human neutrophil antigen 3b (HNA-3b) epitope on the Slc44a2 protein is related to the risk of human venous thrombosis (VT). Mice lacking Slc44a2 showed a substantial reduction in neutrophil recruitment in the inflamed mesenteric venules. Slc44a2/HNA-3a plays an important role in the adhesion and activation of neutrophils in veins under inflammation and specific shearing (80).

### NETs in Pulmonary Embolism

Acute pulmonary embolism (PE), isolated or combined with deep vein thrombosis (DVT), is the main cause of death or hospitalization due to venous thromboembolism (VTE) (81), accounting for 5–10% of deaths in hospitalized patients. The acute all-cause mortality of patients with venous thromboembolism is 6.6% (82). The risk of death after pulmonary embolism is particularly high, 2.1 times higher than that of deep vein thrombosis (DVT) (83).

VTE is related to the release of NETs. Extracellular chromatin and citrulline histone H3 (citH3) have been observed in deep vein thrombosis in patients with VTE (61). Compared with those in healthy controls, NET formation markers in symptomatic patients with VTE were significantly increased (84). The increased formation of NETs reflected by the level of citH3 is positively correlated with impaired fibrinolytic function and may be related to the severity of the disease by enhancing inflammation and the prethrombotic state. A high endogenous thrombin potential (ETP) combined with elevated citH3 levels and prolonged clot lysis time (CLT) are associated with an eight-fold increase in the risk of PE-related death (85, 86). Thrombotic fibrillin clot characteristics and enhanced neutrophil extracellular trap formation are associated with a higher risk of early death in patients with acute PE, which suggests the role of these biomarkers in determining prognosis.

Extracellular DNA in human plasma is also called cell-free DNA (cfDNA) (87–91). The accumulation of cfDNA in the circulation is thought to result from increased cell death and/or activation, impaired cfDNA clearance, and/or decreased endogenous DNase enzyme levels (92). DNA is mainly released by neutrophils through NETosis (93). Circulating cfDNA and nucleosomes are considered surrogate markers of NETs in patients with venous thromboembolism. Elevated levels of circulating nucleosomes and neutrophil elastase/a1-antitrypsin complexes are associated with a threefold higher risk of DVT (65). The plasma cfDNA level is positively correlated with d-dimer and von Willebrand factor levels, as well as with MPO activity, suggesting that neutrophils are the main source of plasma cfDNA in patients with venous thrombosis. Nucleic acid-binding polymers have been shown to prevent thrombosis in mice without increasing the risk of bleeding. These polymers bind to DNA, RNA and inorganic polyphosphate molecules with high affinity and inhibit the activation of the intrinsic coagulation pathway induced by nucleic acids and polyphosphates (94). The complex of extracellular DNA, histones and neutrophil-derived peptides stimulates the inflammatory response by activating pattern recognition receptors on immune cells (95, 96). Circulating extracellular DNA is an independent predictor of mortality in elderly patients with venous thromboembolism (97).

Many substances are currently confirmed to be related to the treatment of thrombosis. DNase I pretreatment degrades NETs and reduces the incidence of thrombosis in wild-type (WT) mice (62, 98). The addition of deoxyribonuclease I to tissue plasminogen activator (tPA) significantly accelerates the dissolution of thrombi and human lung thrombi (99). Glucocorticoids exert important anti-inflammatory effects and regulate the inflammatory immune response in the body. In an infected state, glucocorticoids inhibit the formation of NETs, and as the concentration of glucocorticoid treatment increases, the inhibitory effect becomes more obvious (99). Because NETs are positively charged, unfractionated heparin (UFH) may be a more effective anticoagulant in patients with acute PE. Patients with acute PE presenting lactic acid concentrations >2 mM have a higher possibility of suppressing strong NETosis, and thus UFH may be the first choice (97). Heparin has been shown to bind to histones and prevent histone-mediated cytotoxicity of endothelial cells. *In vivo*, heparin reduced the mortality of aseptic inflammation and sepsis in a mouse model. The protective effect of heparin is not related to its anticoagulant properties (100). Heparin also replaces histones from the chromatin backbone of NETs, thereby destroying the stability of NETs (87). However, recent studies have shown that heparin stimulates NET formation *in vitro*, and the ability of enoxaparin to induce NET production is much lower than that of heparin, while pentosan sodium (fondaparinux) does not induce the formation of NETs (101). The hypothesis of the potential benefits of heparin therapy for patients with PE in real life requires further verification.

## NETs and Cancer-Related Thrombosis

In current research on thrombosis, an increasing number of people are paying attention to the effect of the formation of neutrophil extracellular traps (NETs) on thrombosis, including thrombosis from the automatic venous system and acquired thrombosis (102, 103), such as tumor-related thrombosis and other diseases. The formation of tumor-associated thrombosis may be related to thrombosis derived from the arteriovenous system, but the specific mechanism is not yet fully understood. This may result from the interaction of several mechanisms (104). The risk of deep vein thrombosis (DVT) in patients with cancer is 5–7 times higher than that in healthy people. Tumor-related thrombosis increases the risk of death by 47 times and seriously affects the survival and prognosis of patients with cancer (105). The cause may be a source of cytokines, and tissue damage caused by radiotherapy and chemotherapy causes patients with tumors to usually be in a hypercoagulable state or a prethrombotic state (106). The mechanism of VTE formation (Virchow's triad) is composed of three components: blood flow stagnation, endothelial injury, and a hypercoagulable state. The latter includes abnormalities in the coagulation and fibrinolytic pathways and platelet activation. Tumor-related thrombosis has been roughly divided into direct mechanisms and indirect mechanisms, which are described below (Figure 2C).

### Direct Mechanism

Tumor cells directly induce platelet activation. Activated platelets stimulate tumor cells or normal cells in the tumor microenvironment (TME) to secrete a large amount of thrombo-activating factors, including tissue factor (TF) and collagen exposure. These factors increase vascular permeability and then start the coagulation cascade (107). The expression of podoplanin (PDPN) directly causes platelet activation and aggregation through calcium-dependent lectin-like receptor 2 (CLEC-2) on platelets (108). Tumor cells also secrete platelet agonists, such as ADP and thrombin, which further promote platelet activation through P2Y12 and protease-activated receptor 1/4 (PAR1/4), respectively (109). Phosphatidylserine (PS) on tumor cells also promotes blood coagulation, and PS also serves as a key surface component of the coagulation complex (110). The coagulant secreted by tumor cells has been shown to directly activate the coagulation pathway by activating factor X, stimulating the local TME and even systemic platelet activation and thrombosis. In addition, plasminogen activation inhibitor-1 (PAI-1) is expressed at high levels in tumor cells, inhibiting the process of fibrinolysis (111).

### Indirect Mechanism

Inflammatory cytokines secreted by tumor cells cause platelet activation and promote the procoagulant phenotype of endothelial cells. Platelets also promote tumor metastasis through different mechanisms. Activated platelets protect tumor cells from immune recognition in the circulation, promote tumor cell adhesion, and help tumor cells pass through the blood vessel wall. Platelet α-granules released after platelet activation carry a large amount of growth factors, such as PDGF, VEGF and TGF-β. In the TME, these growth factors are conducive to tumor proliferation, angiogenesis and invasiveness, thereby promoting tumor progression (112). At the same time, these factors cooperate with heparanase and matrix metalloproteinases (MMPs) in the TME to further promote tumor-related thrombosis (113).

Previously, researchers postulated that the blood coagulation process and the immune regulation of neutrophils are two processes that do not interfere with each other, but the latest research shows that they are closely related. Neutrophils infiltrating the TME release cytokines and enzymes, which promote immune thrombosis (114). Studies have shown that NETs cause a prethrombotic state and thrombosis through various mechanisms. NETs cause the release of a large number of histones and proteasomes from neutrophils into the blood. NETs serve as mesh scaffolds to physically capture platelets or interact with NETs. Histones increasingly activate platelets and enhance the adhesion, aggregation and release functions of platelets. These changes ultimately lead to fibrin deposition and red blood cell capture, thereby accelerating the clotting process (115). In addition, growth factors secreted by tumor cells also stimulate neutrophils to release NETs (114). The interaction of NETs and platelets provides a new target for the clinical evaluation and treatment of thrombosis, and effective intervention in the formation of tumor-related thrombosis has important clinical significance.

### Research on NETs and Tumor-Related Thrombi in Animal Models

NETs are present at high levels in a variety of malignant tumors, and the close relationship between NETs and tumor-related thrombosis was first confirmed in animal models (116). In mouse models of breast cancer, non-small cell lung cancer and chronic myelogenous leukemia, neutrophils are more likely to induce the formation of NETs (117). In many different types of malignant tumors, increases in plasma free DNA and circulating NET levels are observed, which are closely related to spontaneous thrombosis (118). Compared with tumor-free mice, tumor-bearing mice had increased venous and arterial thrombosis, and citrulline histone H3 (8) was directly detected in the thrombus. Neutrophils isolated from tumor-bearing mice showed higher H3Cit levels upon *in vitro* stimulation, and NETs formed more readily than normal neutrophils (119). An endotoxin injection into the abdominal cavity of tumor-bearing mice induces the formation of NETs, which in turn induces a prethrombotic state and coagulation dysfunction (47). The accumulation and activation of neutrophils at the site of endothelial injury is considered to be the cause of thrombosis. NETs cause endothelial cell damage. Studies have found that phenyl iodide significantly inhibits this damage, indirectly indicating that endothelial cell damage leads to the formation of thrombi.

Spontaneous DNA-rich thrombi were observed in the lungs of tumor-bearing mice, consistent with the increase in plasma H3Cit and extracellular DNA levels (120). Notably, DNase I pretreatment or depletion of neutrophils completely eliminates arterial thrombosis in tumor-bearing mice and control mice and reduces the size of venous thrombosis in tumor-bearing mice but does not affect venous thrombosis in control mice (8). Based on these studies, DNase I inhibits the formation of NETs or promotes the degradation of NETs, thereby reducing tumor-related thrombosis. Tumor-related neutrophils alter the function and status of host immune cells by producing various chemokines, inflammatory factors and reactive oxygen species. Increased formation and release of NETs were observed when neutrophils were stimulated with tumor-derived granulocyte colony-stimulating factor (G-CSF) (121). Another mechanism by which platelets activate tumor cells to promote the formation of NETs has recently been proposed. Platelets are carriers of tumor-derived exosomes (EVs), which in turn promote the formation of NETs (122). Coincubation of tumor-derived EVs with neutrophils enhanced the formation of NETs, and EVs adhered to the NET complex. The interaction between tumor-derived EVs and neutrophils may lead to tumor-related thrombosis in breast cancer. Similarly, the increase in TF levels in pancreatic tumors accelerates the adhesion of EVs to NETs (118).

### Clinical Research and Application of NETs and Cancer-Related Thrombi

Clinical data have shown that patients with tumors are more susceptible to the formation of NETs, and NETs play a very important role in the process of tumor-related thrombosis. NETs released from neutrophils isolated from patients with tumors exhibit increased thrombin and fibrin production in the plasma of healthy patients (123). Therefore, NETs are observed in the thrombi of patients with tumors. Pancreatic cancer is one of the tumors most prone to thrombosis. Recently, human-derived pancreatic cancer cells (AsPC-1) were shown to induce rapid NET formation and release when cocultured with neutrophils from healthy humans (124). Similarly, the NET release capacity of neutrophils from patients with gastric cancer is also increased compared to that of neutrophils from patients without tumors (125). Both human lung cancer tissues and osteosarcoma tissues display neutrophil necrosis and NETs, which are closely related to the therapeutic effect and prognosis (126). Interestingly, in a clinical study involving 936 patients with newly diagnosed tumors or progression after remission, plasma H3Cit levels were related to the occurrence of VTE in patients with lung cancer and pancreatic cancer but not in patients with other tumors, such as breast cancer and brain cancer. Plasma H3Cit levels were unrelated to VTE in patients with colorectal cancer and prostate cancer (127). We documented the colocalization of H3Cit-positive cells and extracellular H3Cit with extracellular DNA, indicating the presence of NETs in the brain, coronary arteries, and lung microthrombi of patients with stroke presenting with potential tumors. The positive correlation between circulating H3Cit levels and the thrombin-antithrombin complex indicates that NETs may promote the activation of coagulation in patients with cancer. This finding suggests the existence and importance of applying NETs as a predictor of the risk of thromboembolism in patients with lung and pancreatic malignant tumors (128). Peptidylarginine deiminase 4 (PAD4) is expressed at high levels in neutrophils that release NETs. The levels of PAD4 and citrullinated histones reflect the level of NETs in the body (129) and evaluate the tendency of patients with cancer to spontaneously form a thrombus. Tumor-related thrombosis is usually accompanied by an increase in circulating hypercitrullinated neutrophil counts and plasma histone hypercitrullination, but this phenomenon does not occur in healthy patients (8). Histone-DNA complexes are abundant in tumor-associated thrombi, but the plasma levels of circulating nucleosomes and extracellular DNA in patients are correlated with cancer-related stroke and D-dimer levels (130).

Tumors are systemic diseases. This change induces neutrophils to release NETs. Although mechanisms related to tumor-associated thrombosis and the formation of NETs have been proposed, the relationship between this interaction and the formation of NETs remains to be studied. Moreover, further large-sample animal experiments and clinical research support for the use of NETs to predict the sensitivity and specificity of tumor-related thrombosis and the determination of the cutoff value are still needed.

## Conclusion and Perspectives

**Figure 3:**
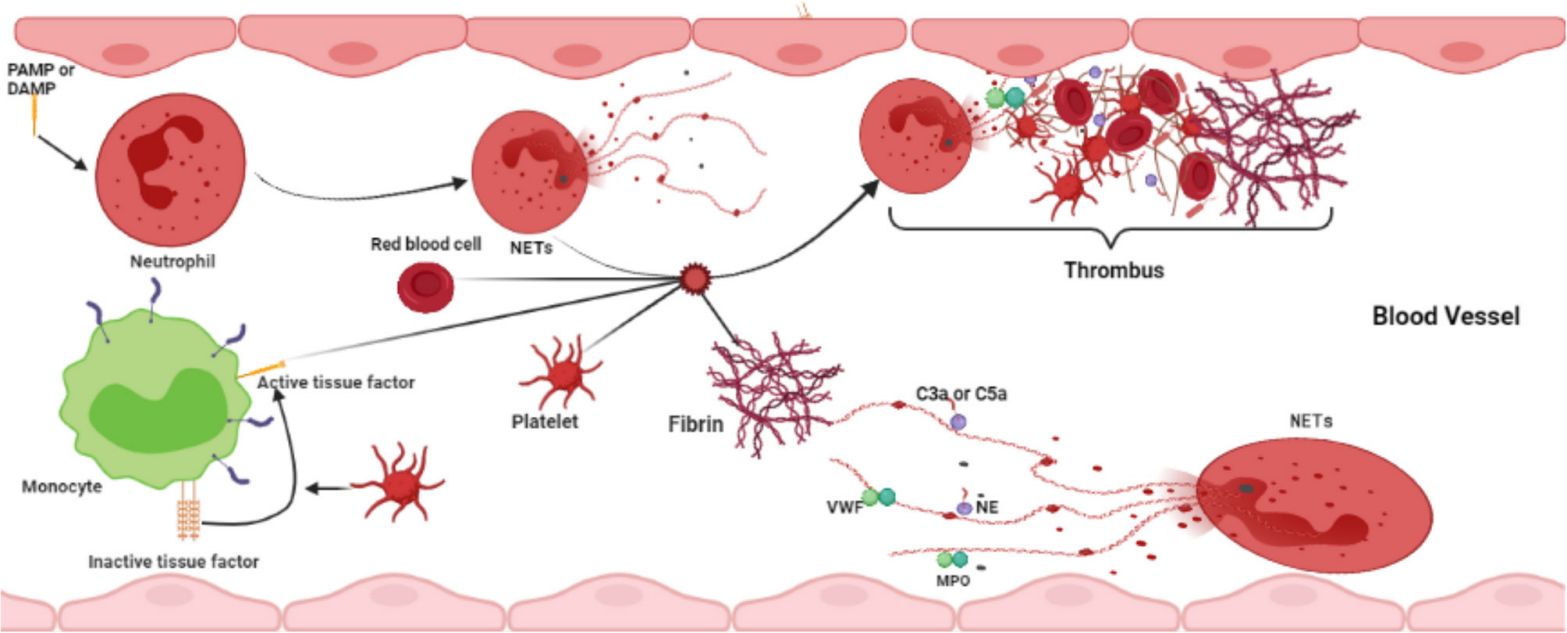
Interaction between NETs and other blood cells in thrombosis formation.

Neutrophil extracellular traps have become undeniable factors in the field of thrombosis and hemostasis. NETs regulate thrombosis through different pathways and are implicated in the pathophysiology of both arterial and venous thrombotic complications (Figure 3). Undoubtedly, future studies will further advance our knowledge of the temporal and spatial processes of neutrophil-driven thrombus formation and maturation. This information will become very valuable for developing novel antithrombotic therapies. Pharmacological disassembly and degradation of NETs in thrombi may enhance acute thrombosis. In addition, strategies preventing the formation of NETs may reduce thrombogenicity, which might be beneficial in thrombosis prevention. Preclinical and clinical studies investigating these new therapeutic opportunities are now needed to fully understand the efficacy and safety of targeting NETs in thrombosis.

## Data Availability Statement

The original contributions presented in the study are included in the article/supplementary material, further inquiries can be directed to the corresponding authors.

## Author Contributions

All authors listed have made a substantial, direct, and intellectual contribution to the work and approved it for publication.

## Funding

This work was supported by the grants of Science and Technology Commission of Shanghai Municipality (20Y11901400) and Pudong Health Committee of Shanghai (PW2020D-13).

## Conflict of Interest

The authors declare that the research was conducted in the absence of any commercial or financial relationships that could be construed as a potential conflict of interest.

## Publisher's Note

All claims expressed in this article are solely those of the authors and do not necessarily represent those of their affiliated organizations, or those of the publisher, the editors and the reviewers. Any product that may be evaluated in this article, or claim that may be made by its manufacturer, is not guaranteed or endorsed by the publisher.
